# The Construction of Good Vulcanite Dentures

**Published:** 1911-11-15

**Authors:** J. H. Prothero

**Affiliations:** Chicago


### THE CONSTRUCTION OF GOOD VULCANITE DENTURES.

BY J. H. PROTHERO, D.D.S., CHICAGO.

It is a deplorable fact that very few dentures are constructed so as to grind and reduce food substances with anything like the same thoroughness that is possible with the normal human masticatory apparatus. The average denture of today is capable of occasionally puncturing morsels of food, but is practically useless as far as grinding is concerned. This is due to the fact that only occlusal conditions are considered and worked out, usually very imperfectly at that, for few attempt to modify the occlusal forms of artificial teeth and fewer still to modify the cusp forms so as to permit the anatomical movements to be performed.

The force required to crush food between teeth with well developed occlusal surfaces when the hinge motion is made use of is much greater than if the lateral movement is employed.

A comparison of the results of grinding versus crushing force is found in Dr. Head's article in the Dental Cosmos, December, 1906. Here he makes use of the findings of Dr. Black's phago-dynamometer as furnishing a fair test of the force required to crush food by direct thrust and compares these results with experiments conducted with a human masticatory apparatus in which the food was subjected to the grinding motion.

The following is the table referred to:

| Meats. | Head. | Black. |
|---|---|---|
| Corned beef | 18–22 | 30–35 |
| Tongue | 1–2 | 3–5 |
| Tenderloin of beef, very tender | 8–9 | 35–40 |
| Round of beefsteak, tough | 38–42 | 60–80 |
| Roast beef | 20–35 | 35–50 |
| Boiled ham | 10–14 | 40–60 |
| Pork chop | 25–30 | 20–25 |
| Roast Veal | 16 | 35–40 |
| Average | 17–20 | 32¼–41⅞ |
| **Vegetables.** | | |
| Raw cabbage | 16 | 40–60 |
| Head lettuce | 8 | 25–30 |
| Radish, whole brake at | 20–25 | 20–25 |
| Radish, pieces pulverized at | 10–15 | 35–40 |
| Average | 12½–16 | 30–38¾ |

Is any further argument necessary to convince the prosthetist that the greatest usefulness with the least expenditure of force is developed in teeth occluded anatomically. If so as an additional argument let it be borne in mind that anatomically arranged teeth balance. In other words there is no idle side, for while food is being reduced and ground on one side the dentures on the opposite side are supporting each other in smooth gliding contact. System in that, isn't there. Might as well expect an eight-wheel ordinary locomotive to run on a mono-rail and maintain its equilibrium as that unbalanced dentures will remain seated under active lateral masticatory effort.

A contributory as well as a frequent primal cause of poor adaptation lies with the impression. Unless the impression is manipulated skillfully and removed from the model immediately on the setting of the plaster a warped as well as an expanded model results. The change is slight, it is true, and a careless prosthetist might not discover any fault of adaptation in a denture produced from such model because the careless prosthetist never has a case of excellent adaptation.

I say this without fear of contradiction for adaptation does not happen, it is produced and if one gets fair adaptation in favorable cases without effort better could be secured if striven for. Experience has led me to discard plaster of Paris for impression work whenever possible.

Three classes of cases present however, where this material is positively indicated: First, where any of the natural teeth are present. Second, where there are under cut surfaces opposed to each other. Third, where the ridge is flabby and will be readily distorted or bent out of shape. In all other cases a modelling compound that becomes plastic at very low temperature and hardens quickly can be used to best advantage. Two such compounds are on the market to my knowledge. Stent's Compound made by Ash & Sons, and Perfection Compound, made in Detroit. But little expansive or contractile change is noticeable in either of these varieties and what little may occur is eliminated by subsequent procedures not possible to employ with plaster.

Method of manipulation for upper impressions is as follows: Select a tray of appropriate size, but long enough to cover all of the hard palate if possible. Place compound and introduce in mouth pressing well up to position. Conform the labial and buccal surfaces of border by external pressure. In most cases pass the middle fingers of both hands back in central palatine vault and lift the compound in this location up against the soft palate, holding the material in position until fairly well hardened (the tray being held with the index fingers). Remove and chill. The impression will come away easily, as the adaptation is not yet developed. Trim off excessive surplus, round away the angles resulting from the cutting and remove all chip debris.

Now invert over a small gas or alcohol flame and soften the impressed surfaces to the depth of 1 to $1\frac{1}{2}$ mm. and quickly return to the mouth. The chair should be tipped back so that the operator can stand directly behind the patient and when the impression is again seated a firm steady pressure of from 15 to 30 pounds is maintained upon the tray for three or four minutes to bring about a re-adjustment of the softened film of compound. Under the pressure the compound moves from the hard to the softer areas, in fact it is crowded onto and compresses them.

On removal the impression is again chilled and reheated if necessary and the process repeated. Patients frequently complain bitterly of the discomfort experienced in removal of the impression which in some few cases observed, owing to the almost complete exclusion of air from between the contact surfaces indicated that nearly a perfect vacuum had been produced. Lower impressions are treated in a similar manner, but on account of the decreased area involved, less effort will be required for dislodgment than in upper cases.

Upper impressions should be relieved in the central palatine portion to a slight extent, say $\frac{1}{2}$ mm. for even with the compression, of soft tissues carried out in the impression, under use the denture is inclined in time to settle and rock.

In lower cases the deepest part of the impression or that portion which reproduces the crest of the ridge is sometimes slightly scraped from about the region of the second molar to the corresponding tooth on the opposite side. By so treating these cases the crest of the border is not impinged on when the lower denture is under load and in addition this treatment permits the peripheral margins of the denture to settle firmly into and against the soft tissues thereby excluding the air. In many cases a partial vacuum is developed which materially adds to the stability.

A few words on models will be applicable at this time. All plasters, good, bad and indifferent, expand in setting, some more, others less, all variable, depending on conditions and how manipulated. Moderately coarse plaster, medium setting as to time, good hardness when set is preferable to the finely ground quick setting varieties for model work because of less expansion.

A moderately thick mix should be made, the mass stirred slightly and introduced into the impression as quickly as possible. As soon as hard enough to handle, remove the tray and impression to prevent warpage.

Trim the labial and buccal surfaces somewhat inside of peripheral outline with a discoid instrument to a very slight depth to compensate for general expansion. This trimming raises an imperceptible bead on these surfaces and if properly located will not be obliterated by subsequent peripheral trimming frequently necessary to relieve irritation. A plane should be scraped across the distal part of the model usually about one-eighth inch wide and meeting the buccal beads at the tuberosities. This area should be tapered from nothing anterially to $\frac{1}{2}$ mm. in depth at the distal margin and forms at this point the line or distal termination for the denture.

The beading on the sides of the model compensates for general expansion of the plaster, the line across the distal insures the base plate being imbedded in the tissues and guards against the ingress of air in this most vulnerable area.

The construction of base plates on which to build rims of wax for determining facial contour, length of teth and for bite purposes, locating the condyle ends, taking the bite, use of the face bow, and mounting the models on the frame have been well described in previously published articles and it is not necessary to take up your time by such repetition. Clinics will be given to make the various steps clear when at the same time the registering of the condyle path will be explained.

The next important step in logical order will be the preliminary grinding of the teeth selected for a given case. It will be found a most convenient and time-saving method to modify the occlusal surfaces of all the bicuspids and molars even in the best forms of teeth now supplied us by the manufacturers if the greatest efficiency is desired.

A moderately fine grit carborundum stone $\frac{3}{8}$ of an inch face, 3 inches in diameter is a convenient size to use. An emery truing device should be applied to each angle and two faces developed which meet in the center of an angle of about 120 degrees.

The central groove of each tooth is applied to this angle, while the two surfaces cutting buccally and lingually knock off the high points and develop somewhat flattened planes where only marble like surfaces existed. Teeth properly treated in this manner will still retain most of their characteristic surface markings and when occluded will fit together and work like mill stones.

In full cases the upper teeth are arranged first, the wax rim indicating their correct position. The second bicuspid on one side is the first in the lower arch to be placed, because by beginning with a tooth the varying occlusal planes of which are received by corresponding opposing planes the broadest contact areas will be utilized. The placing of this tooth in correct occlusal position, however, does not prove its correctness under action and it should be tested by subjecting it to lateral frame movements. Frequently the tooth itself or one or both opposite may require slight rotation to develop free clearance, but such changes are easily and quickly made, and if the teeth are firmly fixed in hard wax, will not often require re-adjustment.

The molars and first bicuspid are then set, each being tested as was the preceding one, then the corresponding opposite teeth are similarly placed and proven.

The cuspids, laterals and centrals are next adjusted in the order mentioned and if too wide or narrow, the discrepancy is overcome by substitution or reduced by grinding.

Finally the balancing point is worked out usually by tipping the two last molars, the upper backward and the lower forward until their occlusal planes are parallel with the condyle path. This can readily be determined by lateral frame movements and when correct the case is ready for waxing and trial in the mouth.

A hard variety of base plate wax should be used in constructing the rims and for waxing the teeth in position in order that the teeth will not move under stress in the mouth.

The patient should be instructed to exercise the mandible freely in lateral and protrusive movements, the operator at the same time holding the lips and cheeks away and observing the relationship of the teeth in the various positions. Frequently a slip of carbon paper placed between the occlusal surfaces will disclose high points not otherwise noticeable and these can be corrected with a small engine stone.

The principal point to observe, however, is whether the balancing points of contact have been developed. The high point can be corrected in the finished case, but not so with the balancing points. These must be secured by the parelleling of the occlusal planes of the last molars with the condyle path as before stated. When these several points are worked out correctly the cases are ready for flasking.

A case in which a perfect impression has been secured, a model obtained from that and corrected as perfectly as possible, the anatomical condition worked out accurately and the flasking properly accomplished may be ruined or the adaptation seriously impaired in the packing and vulcanizing.

The cause briefly stated is due to the instability of plaster. Pulverized dental plaster has the formula $CaSO4$ $\frac{1}{2}$ $H2O$. The calcined powder when examined under the microscope is seen to consist not wholly of amorphous granules, but with broken parallel sided crystals of widely varying sizes interspersed. When mixed with water the half hydrate begins immediately to take up water and the di-hydrate is formed, formula CaSO4 2(H2O), the process going on for thirty-six hours or longer. If some freshly mixed plaster is placed on a slide and a cover glass pressed over it with a sliding motion so as to thin out the mass and render it transparent, when examined under the microscope tufts of needle-shaped crystals will be seen to pile up on each other and as the setting process goes on other crystals will gradually be attracted to and form upon the mass already grouped. These crystals are long delicate and needle-shaped and are grouped without order, lying very much like a bunch of disorderly jack straws with many open irregular spaces between.

These spaces may or may not become partially filled by the more slowly setting finer particles which crystalize in shorter needles later on.

In any event a plaster model which appears hard and resistant is composed of innumerable delicate crystals so grouped as to leave an intricate network of open spaces permeating the whole mass. Crystals of the character described will withstand stress up to a certain point, but will be instantly shattered when the modulus of resistance is reached.

If our eyes possessed microscopic power or the crystals of paster were of microscopic size, we could see a most terrific convulsion occurring during the closing of an overpacked flask. Crystals shattering and flying in all directions, others bearing their load up to the modulus limit and merely breaking and with others again maintaining their integrity and form depending on the force exerted upon them. Each broken crystal on the face of a model paves the way for a misfit denture.

Assuming the modulus of resistance to be 5, the ratio of force ordinarily exerted in closing a flask ranges from 2 below to 35 in excess of the modulus. Is it any wonder that misfits occur?

How can this danger be obviated?

Use dry heat in warming the waxed case before separating. When open, remove all wax from the matrix that can possibly be taken away with instruments. A fine stream of boiling water directed into the matrix will almost instantly remove the residue. No water need be applied to the model since, if proper care has been taken it will be free from wax.

The matrix is heated slowly over a flame first to drive off moisture and secondly to aid in the packing of the rubber.

The old method of gauging the amount of wax in a water glass and using a like bulk of rubber in the packing is an easy and rapid and accurate plan to follow.

When packed, a piece of muslin from the rubber, freed from its starch by washing and wrung dry as possible is spread over the packed case. The flask is then closed as close as possible with the fingers and the bolts inserted, tightening the nuts only slightly.

Dry heat is then applied, the flask being turned at frequent intervals to present the various surfaces to the flame. When well heated the wrench is used to tighten, applying not to exceed 5 pounds on the end of the wrench handle.

If necessary, reheat to obviate undue stress on the model. When closed, the bolts are removed and flask separated. Moisten the muslin with water applied with a pellet of cotton, when it will peel away from the rubber without difficulty.

Examine the packed matrix with a blunt, round-pointed instrument, pressing here and there to see that the mass is solid. A slight excess should show at the margins, although this is not imperative.

If deficient, add enough to make up the required amount and close the flask.

Place in a vulcanizer in the bottom of which is a block of wood, iron or preferably zinc about $\frac{3}{4}$ of an inch thick and $2\frac{1}{2}$ inches in diameter. This leaves a space between the walls of the vulcanizer and the block amply sufficient for the water which should never cover the block. A cubic inch of water will make a cubic foot of steam, so don't be afraid of burning your case unless the vulcanizer leaks. As a matter of fact a teaspoonful of water is sufficient for a vulcanizer full of flasks.

It will be found that if the block on which the flask rests is of zinc, that the hydrogen sulphide which forms during vulcanization will attack it instead of the flasks or vulcanizer walls, they remaining clean while the zinc is gradually destroyed.

I have been taken to task for recommending this method, the claim being made that the presence of the zinc will cause porosity in the vulcanite.

We have since made accurate records of over 10,000 cases in our school laboratories and in that number but three cases came out porous. These cases were very thick, which fact is self-explanatory.

The best quality, greatest density and most elastic properties are developed in rubber with less risk of porosity occurring if vulcanization is carried on at 290 degrees or lower for a time sufficient to harden, rather than at a higher temperature for a shorter period.

This method of vulcanization insures a dry model in which the plaster crystals are far more resistant to stress than when damp or saturated, during the closing of the flask and further insures the maintenance of the model in a comparatively dry condition in the early stages of vulcanization, or during the time when some pressure is exerted on the face of the model by the slight excess of rubber before hardening has occurred.

The finishing of a case is accomplished along the prevalent lines. The palatine and border surfaces however should be finished with pumice stone and a stiff brush wheel until as smooth as any other surface of the denture to prevent the accumulation of mucous plaques and debris. No fear need be entertained of interfering with the adaptation if ordinary care is exercised. The regrinding has been thoroughly explained by Dr. Pritchett and some further details will be enlarged upon in showing the slides relating to this subject. It is a regrettable fact that in some schools outside of Chicago the teaching of anatomical occlusion is sadly neglected. In some cases, in fact, the teachers say it is a delusion and a snare. No man with brains who ever tried the system can make such a statement. If true then the Almighty blundered onto a happy system without realizing it. What is that little Arab Maxim: "He that knows not and knows not that he knows not is a fool."

Let me enjoin the members of our society with all the ardor I can command and with a confidence and a certainty based on practical experience, to put into practice the system here outlined. The results I am sure will be an uplift in the field of prosthesis that will redound to the credit of the profession and the benefit of humanity. *Dental Review*, November, 1911.

